# Supplementary material for: Gain-of-Function p53N236S Mutation Drives the Bypassing of HRasV12-Induced Cellular Senescence via PGC–1α
Source: Int J Mol Sci. 2023 Feb 14;24(4):3790. doi: 10.3390/ijms24043790 (PMC9960896; doi:10.3390/ijms24043790)
Supplement: Supplementary file 1 [file ijms-24-03790-s001.zip › ijms-2086684 supplementary.pdf]

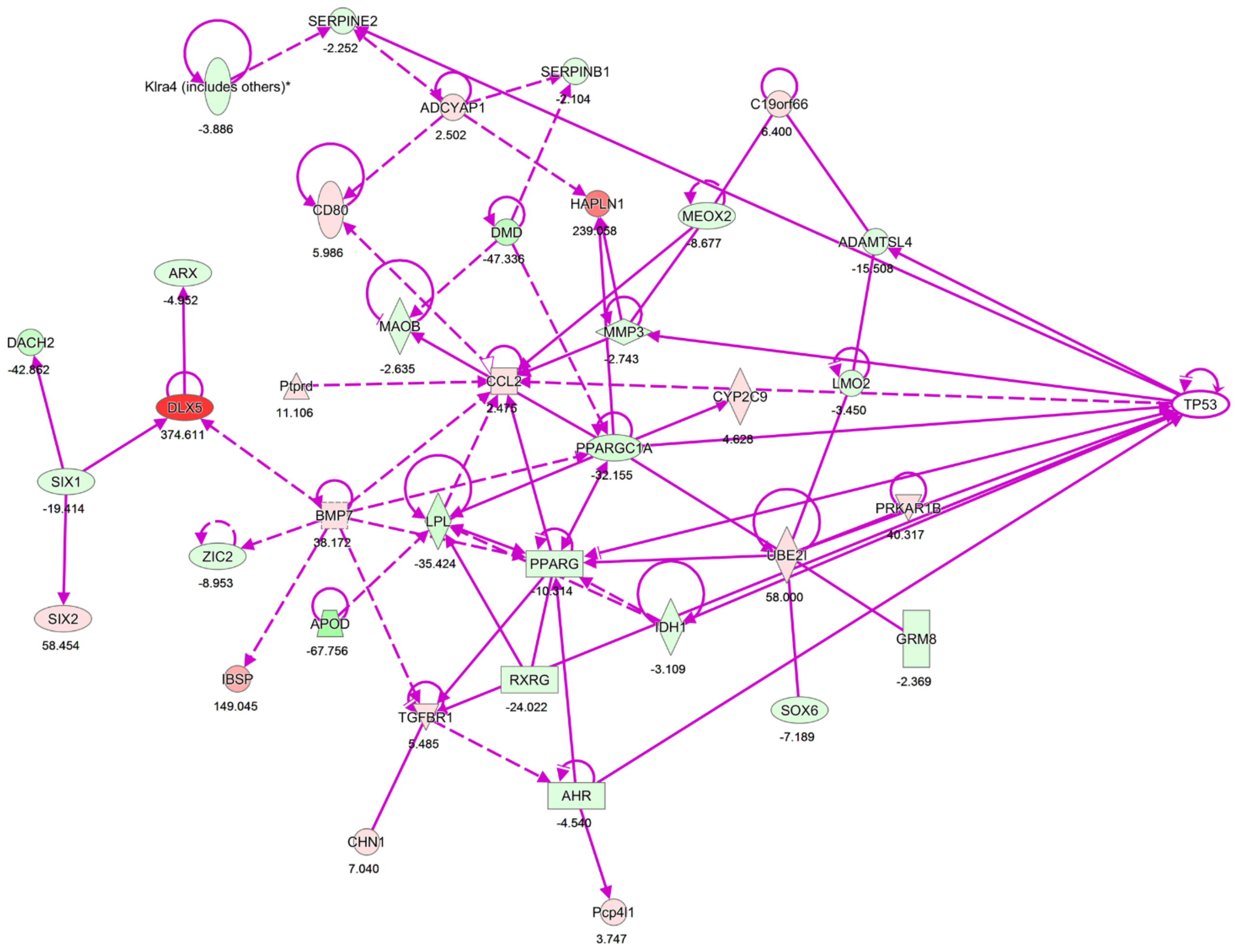

Figure S1. Genes obtained through both the ChIP-on-ChIP and microarray datasets were mapped to a protein interaction network with the central points set to *TP53*.

A

```
target2geo    --acagaatgggcaaatctaggaagggctgtgggttttcccccttggtctgaattaaga
F10           TTACAGAAGGTCCAAATTTAGAAAGGGTAGGGGGTTTTACCCCTTTGGTCTATATAAGAA
                ****** *  *****.***.*****. * ******* ************. ** *..*

target2geo    atccaaactgttcctacgccacggttatgcactgggaagccaaaggcaactctgaccact
F10           GGCCAAAGGGTTCGAACCCTTCGTTAATCCCGGGGGAACCCAAGGCCAATTAAGCCAAGG
              . *****  ****  ** *. ** * ** *   ***** ****.* ***.*  * * *

target2geo    gttaagcagtaggaagggttcttactagagacggctcttctgcctcctgaggggagggg
F10           GTAAACCAGTAGGAAGGG-TAGGAATAGAGACGGCTCTTCTGCCTCCTGAGGGGGAGGGG
              ** ** ************ *   * ***********************************

target2geo    tgccgtcaggcatggaggaaggactggcctcgttgtcagtggtcacggctccatctgtca
F10           TGCCGTCAGGCATGAGGAAGG-----------------------ACGCCTCC--------
              **************..*.*.*                       *** ****

target2geo    gtgcatcaa
F10           -TGCGTCCC
               ***.**
```

B

```
target4geo    ---------------------------------------------ctccccatacatcag
C11           CCATACATCAGTCAGACATGAAGATCAGTCAAGCCGCTGCCGTCTCTCCCCATACATCAG
                                                           ***************

target4geo    tcagacatgaagatcagtcaagccgctgccgtcagagagaagctcatttctccaggggag
C11           TCAGACATGAAGATCAGTCAAACCGCTGCCGTCAGAGAGAAGTTCATTTTTCCAGGGGAG
              *********************.********************.******.**********

target4geo    aggcttacctcaaatatgttcgcaggctcattgttgtactggttggatatgatttccgat
C11           AGGGTTCCATCAAATATGTTTGCAGGCTCATTGTTGTACTGGTTGGATATGATTTCCGAT
              *** ** * ***********.***************************************

target4geo    tggtcgctacaccacttcaatccacccagaaagctgtctgtatccaagtcattcacatca
C11           TGGTCGCTACACCACTTCAATCCACCCAGAAAGCTGTCT-TATCCAG-------------
              *************************************** ******.

target4geo    agttcagaaaggtcaagttcaggaagatctgg
C11           -------------CATGCTGAGGCAG-TACAA
                           ** *.* *** ** * ...
```

Figure S2. The sequences of *PGC-1α*-knockdown clones (F10 Target2 and C11 Target4). The sgRNA sequence is marked in blue.

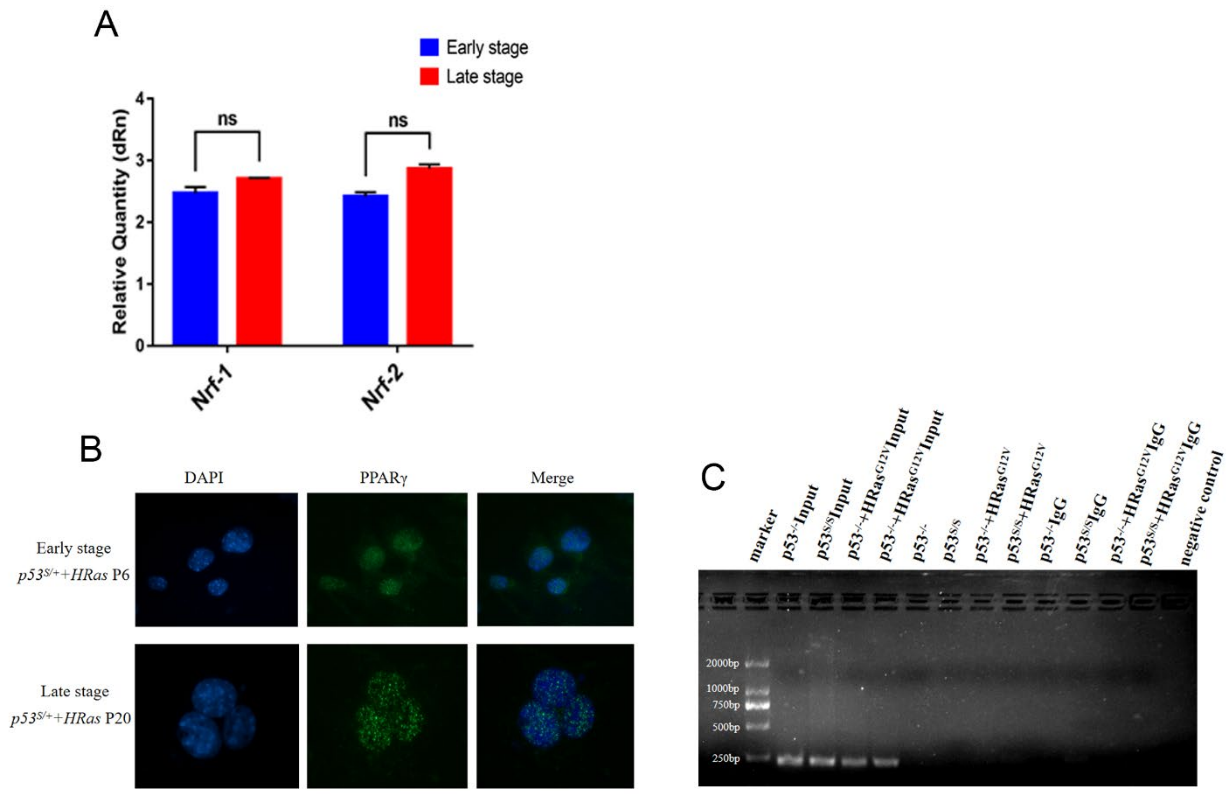

Figure S3. A: Real-time PCR was used to measure the expression levels of *Nrf-1* and *Nrf-2* in ES and LS cells. **B:** Immunostaining for PPARγ in ES and LS cells. **C:** A ChIP assay was performed to determine the ability of p53S to bind to the PPARγ promoter.
