# Supplementary figures and images for: Gain-of-Function p53N236S Mutation Drives the Bypassing of HRasV12-Induced Cellular Senescence via PGC–1α
Source: Int J Mol Sci. 2023 Feb 14;24(4):3790. doi: 10.3390/ijms24043790 (PMC9960896; doi:10.3390/ijms24043790)

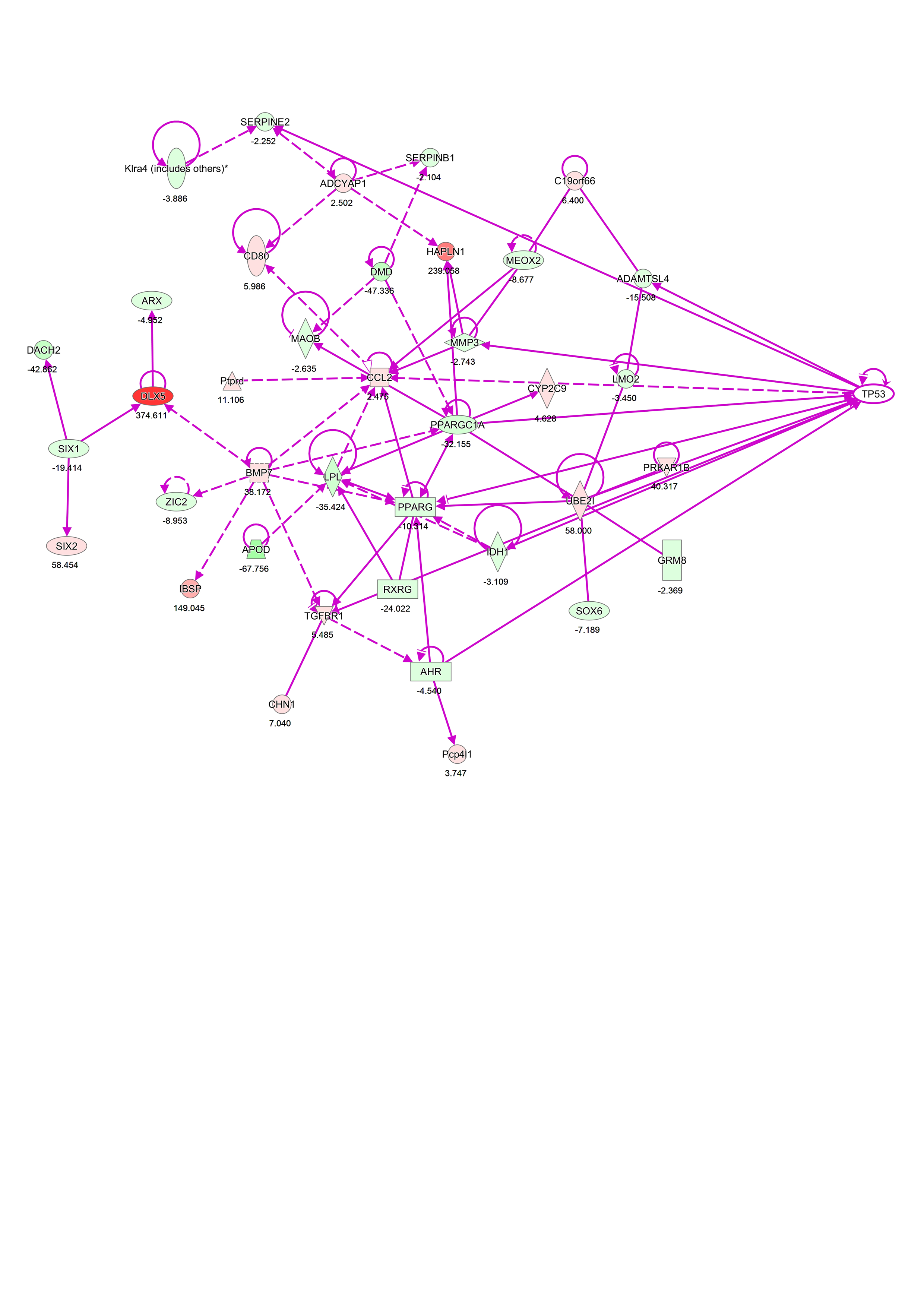

Supplement: Supplementary file 1 [file ijms-24-03790-s001.zip › Supplementary Figures/sup figure1.tif]

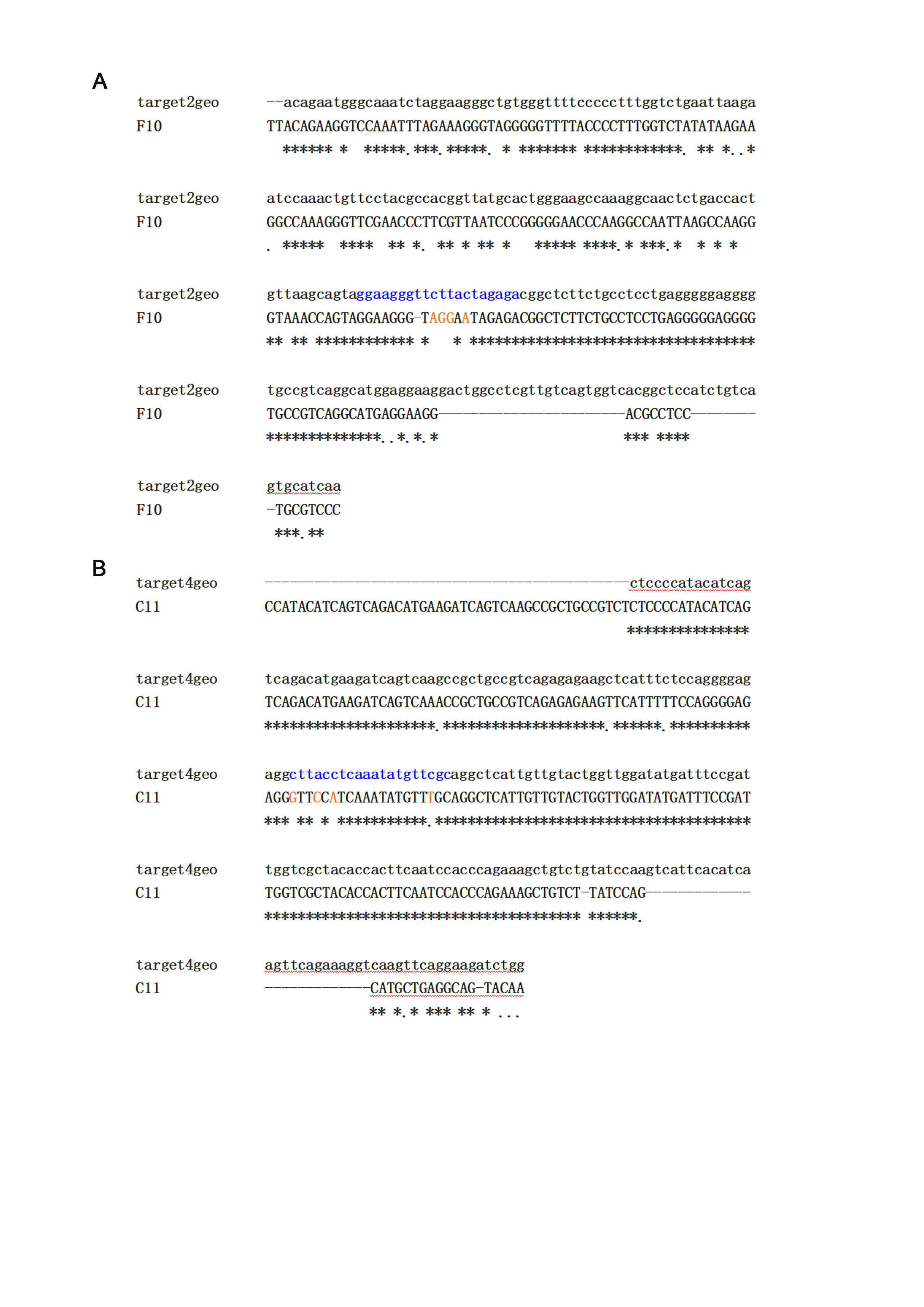

Supplement: Supplementary file 1 [file ijms-24-03790-s001.zip › Supplementary Figures/sup figure2.tif]

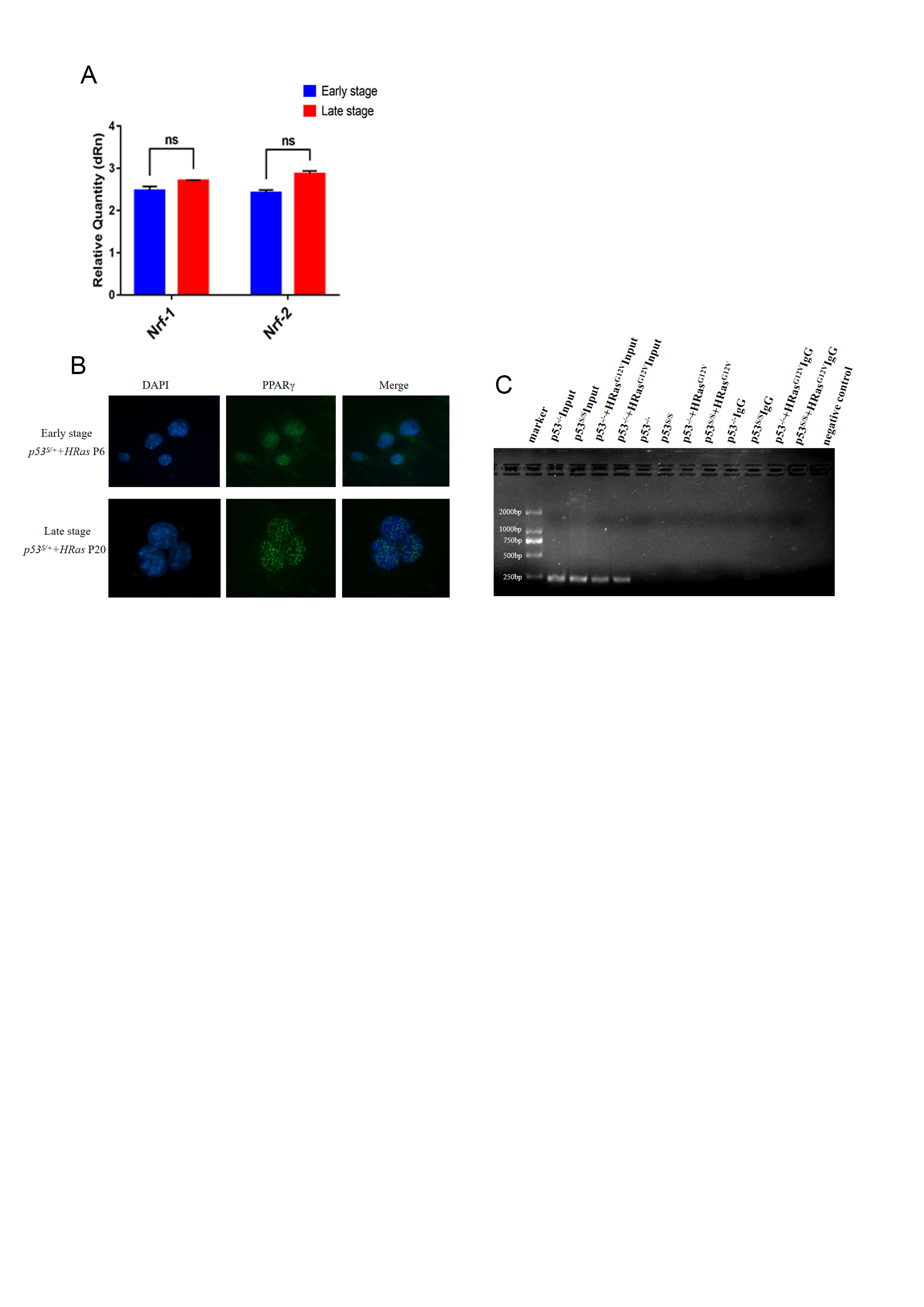

Supplement: Supplementary file 1 [file ijms-24-03790-s001.zip › Supplementary Figures/sup figure3.tif]
